# Uncovering blind spots in urban carbon management: the role of consumption-based carbon accounting in Bristol, UK

**DOI:** 10.1007/s10113-017-1112-x

**Published:** 2017-02-03

**Authors:** Joel Millward-Hopkins, Andrew Gouldson, Kate Scott, John Barrett, Andrew Sudmant

**Affiliations:** Sustainability Research Institute, University of Leeds, Leeds, UK

**Keywords:** Carbon footprint, Cities, Climate policy, Consumption-based emissions, Mitigation, Sustainable consumption

## Abstract

The rapid urbanisation of the twentieth century, along with the spread of high-consumption urban lifestyles, has led to cities becoming the dominant drivers of global anthropogenic greenhouse gas emissions. Reducing these impacts is crucial, but production-based frameworks of carbon measurement and mitigation—which encompass only a limited part of cities’ carbon footprints—are much more developed and widely applied than consumption-based approaches that consider the embedded carbon effectively imported into a city. Frequently, therefore, cities are left blind to the importance of their wider consumption-related climate impacts, while at the same time left lacking effective tools to reduce them. To explore the relevance of these issues, we implement methodologies for assessing production- and consumption-based emissions at the city-level and estimate the associated emissions trajectories for Bristol, a major UK city, from 2000 to 2035. We develop mitigation scenarios targeted at reducing the former, considering potential energy, carbon and financial savings in each case. We then compare these mitigation potentials with local government ambitions and Bristol’s consumption-based emissions trajectory. Our results suggest that the city’s consumption-based emissions are three times the production-based emissions, largely due to the impacts of imported food and drink. We find that low-carbon investments of circa £3 billion could reduce production-based emissions by 25% in 2035. However, we also find that this represents <10% of Bristol’s forecast consumption-based emissions for 2035 and is approximately equal to the mitigation achievable by eliminating the city’s current levels of food waste. Such observations suggest that incorporating consumption-based emission statistics into cities’ accounting and decision-making processes could uncover largely unrecognised opportunities for mitigation that are likely to be essential for achieving deep decarbonisation.

**Electronic supplementary material:**

The online version of this article (doi:10.1007/s10113-017-1112-x) contains supplementary material, which is available to authorized users.

## Introduction

The rapid urbanisation of the twentieth century is set to continue through the twenty-first century. Nearly four billion people now live in cities, and this is forecast to rise to over six billion (67% of the forecast world population) by 2050 as urban populations—especially in the developing world—continue to grow (UN 2014).

With this majority share of the global population, it is unsurprising that urban areas are now responsible for a substantial share of anthropogenic environmental impacts. As a fraction of global levels, cities account, directly, for approximately two-thirds to three-quarters of both final energy use and energy-related CO_2_ emissions (Grubler et al. 2012; Kennedy et al. 2015). And when the indirect environmental impacts of cities due to consumption of energy, goods and services are considered, including impacts arising throughout global supply chains, the role of cities appears even more significant (Seto et al. 2014).

For cities, the issue of emissions embedded in imported goods is of particular significance. Over the past decades, an increase in the volume and structure of international trade has enabled an increasing share of production activities, and their associated emissions, to be transferred outside the city (or country) of consumption (Peters et al. 2011). The idea that high-density urban living can enable low-carbon living has gained much traction in recent decades, but evidence from a consumption-based perspective does not support this idea, rather, the primary drivers appear to be income levels and household size (Heinonen et al. 2013). Studies have found that in developed countries such as the UK, when the impacts of imported goods and services are taken into account, emissions are rising even though production-based emissions have been falling (Barrett et al. 2013) such that consumption-based CO_2_ emissions are around twice the level of production-based emissions (Minx et al. 2013). But such trends are not confined to post-industrial economies such as the UK. Even in China—a net exporter of emissions (Chen et al. 2016b; Peters et al. 2012)—cities have been found to have consumption-based emissions that far exceed their production-based emissions (Feng et al. 2014). Moreover, in the cities of lower and middle income countries, in which the majority of the growth in urban population is expected to occur in the coming decades, both per-capita energy use (Grubler et al. 2012) and consumption-based carbon footprints (Guan et al. 2008; Minx et al. 2011) are typically much higher than national averages, the latter substantially so.

In response to the challenge of the rising carbon emissions of urban areas, there is now a surge of research focused upon the global mitigation potential of cities. High-level estimates of potential mitigation suggest that actions throughout the world’s urban areas could reduce their direct, production-based CO_2_ emissions by 10–25% (Creutzig et al. 2015; Erickson and Tempest 2014; Gouldson et al. 2015). Other research suggests that deeper emissions reductions could be achieved by encouraging more compact cities in which high population concentrations may allow for human material needs and wants to be met more efficiently (Creutzig et al. 2015).

Motivated by the mitigation opportunities underlined by such research, various political initiatives have also been developed to help cities work towards achieving these reductions. The Global Protocol for Community-Scale Greenhouse Gas Emission Inventories (GPC) offers a standard framework for cities to follow to report their emissions (WRI 2014), while networks such as C40 bring cities together to measure their emissions, set targets and collaborate and share knowledge to meet these. Using the GPC framework, many of the 80+ cities in the C40 network—which together account for 25% of global GDP—have reported the sources and magnitudes of their current carbon emissions. However, such initiatives are in a relatively early stage of development. Currently, few of the C40 cities have made future projections of their production-based emissions; generally, only 1 year or historical time series estimates exist. Fewer still have undertaken comprehensive environmental and economic appraisals of low-carbon measures to estimate city-scale, production-side mitigation potential, as reported, for example, in Gouldson et al. (2015) and a limited number of other studies in the grey literature (Deloitte 2008; McKinsey 2008). This is despite the fact that such mitigation pathways are becoming increasingly attractive: in addition to evidence that actions can yield economic benefits (Gouldson et al. 2015), the local co-benefits, particularly relating to air pollution and human health, are increasingly well understood (West et al. 2013).

Arguably, however, the most significant issue with the current mitigation strategies of cities is the relatively narrow focus on production-side emissions reductions and hence the absence of a comprehensive account of the carbon associated with cities’ full consumption of energy, goods and services. Emissions monitoring and reduction targets reported through C40, and independently from numerous other cities, are currently focused upon production-based emissions with very few exceptions (SEI 2012). Further, although there are now an increasing number of academic studies measuring consumption-based emissions at the city-scale (see Wiedmann et al. 2015 for a useful summary), future projections, such as those we undertake here or those reported in Straatman et al. (2015), remain extremely rare in the literature.

In summary, a substantial and increasing proportions of cities’ carbon footprints remain largely absent from their local emissions accounts and reduction targets, leaving the ability to reduce these footprints dependent upon (potentially non-existing) production-based mitigation strategies in other regions (Scott and Barrett 2015). Considering the dominant and rapidly increasing contribution of cities activities to global anthropogenic emissions, the absence of consumption-based emissions from local government’s mitigation strategies appears a significant global issue. We are not suggesting that cities must take responsibility for these emissions as such, but demonstrating that cities could have some level of influence over some of the emissions produced outside their boundaries. Accounting methods have developed alternative allocation schemes in which emissions from infrastructure serving the city are included (e.g. electricity supply and rail networks), to a consumption-based approach which goes beyond infrastructure to all goods and services serving a city’s residents and government (Ramaswami and Chavez 2013).

Besides the ethical argument that high consumers should take responsibility for their consumption (Kokoni and Skea 2014), proposals have been suggested where the mitigation responsibility is shared between producers and consumers (Afionis et al. 2016). Responsibility can be apportioned depending on the benefit obtained by each actor along the supply chain or by other social and economic indicators such as average income. Under this approach, there needs to be an understanding of both the production- and consumption-impact of cities, but also the degree to which a city can exercise influence over the consumption behaviour of its citizens and firms will, to some extent, depend on its political ideology and its governance capacities (Kramers et al. 2013).

In this paper, therefore, we develop and apply different methods for carbon accounting at the city-scale and undertake assessments of the associated mitigation potentials, in order to offer an insight into how local mitigation strategies may be focused and accelerated to help address the substantial, and rapidly growing, issue of urban carbon emissions. We first describe and apply a methodology to estimate current and future production-based emissions at the city-level, projecting forward to 2035, using the city of Bristol in the UK as a case study. We then do the same with a methodology for evaluating options for reducing production-based emissions (Gouldson et al. 2015). We analyse both the energy saving potential and associated economic costs and benefits of the mitigation options, formulating scenarios with different levels of ambition based upon economic considerations. Subsequently, utilising methods and data of previous researchers (Barrett et al. 2013; Lenzen et al. 2013; Minx et al. 2013), we compile a historical baseline for the city’s consumption-based emissions, again projecting this forward to 2035. These projections allow us to explore the potential impact that the city’s current ambitions for reducing production-based emissions may have upon its wider, consumption-based, carbon footprint, while also identifying the sectors driving this footprint. We find that even a full deployment of low-carbon measures to reduce the city’s production-based emissions is likely to have a relatively modest impact upon its consumption-based footprint. But we argue that this could be as much an opportunity as a challenge: incorporating consumption-based mitigation into decision-making processes may open up opportunities for emissions reductions that can be achieved more effectively and efficiently than a continuing pursuit of mitigation focused only on the production-side.

## Methodology

### Production-based emissions: BAU

The first stage of the method involves developing a baseline, business-as-usual (BAU) trajectory for production-based (PB) emissions at the city-scale, i.e. the carbon emitted directly within the city’s boundaries and indirectly via electricity use. Our accounting boundaries correspond to scope 1 and 2 emissions, respectively, of the GPC framework (WRI 2014), but do not incorporate the impacts of other essential city infrastructure requirements—e.g. those relating to gas, transport fuels and water—that are included in the *Community*-*Wide Infrastructure Footprint* of Chavez and Ramaswami (2013). We focus on all greenhouse gases, measured as CO_2_e.

To develop a BAU trajectory, we start with historical city-scale emissions data and project these forward by utilising (1) city-level population projections and (2) national-level projections for energy and emissions. Trends in Bristol’s emissions over the period 2005–2012 closely match those occurring at the national-level. First, we match the national-level emitting sectors to the city-level sectors (*domestic*, *transport*, *industry and commerce*, and *electricity*),^1^ aggregating national-level sectors into clusters where necessary. Second, we calculate growth rates in *per*-*capita* emissions from these national-level sectors/clusters. Using these growth rates, we then take the 2012 city-level, per-capita emissions for each sector and project these forward to 2035. Finally, we aggregate these projections into total emissions using the city’s population projections. For the UK, all these data are freely available through the government’s open data site (https://data.gov.uk). Further details describing data and methodology can be found in our supplementary information (SI) and in Gouldson and Millward-Hopkins (2016).

UK-level projections for energy and emissions are available for various scenarios with different energy prices, decarbonisation paths and policy ambitions. These permit us to compile a number of baselines for Bristol relating to nine permutations of central/low/high prices and central/limited/high decarbonisation. While we focus upon the central forecasts of energy prices and decarbonisation for the BAU case, these baselines highlight the sensitivity of our results to these assumptions.

### Production-based emissions: mitigation scenarios

Next we explore strategies to mitigate city-level PB emissions by considering energy efficiency measures and small-scale renewables that could be deployed in the domestic, commercial, industrial and transport sectors. These measures range from improved insulation and appliances in domestic and commercial buildings, through more efficient control systems for industrial applications, to expanded local rail and bus services and increasing numbers of hybrid vehicles. For each sector, we first identify a range of applicable measures and then we assess their investment costs, energy savings and city-wide deployment potentials. A full description of our data sources and assumptions regarding these measures and their deployment, and a summary of our economic analysis for ≈150 measures, are included in the SI and reported in Gouldson and Millward-Hopkins (2016).

Much of the cost and savings data we use are applicable throughout the UK, while deployment potentials must be made specific to the particular city being studied. However, the methods we use for the latter are applicable across the UK and wherever else similar data are available. Transport is the main exception to these generalisations, being reliant upon extensive locally specific data.

We then integrate these cost, savings and deployment data to estimate annual, city-wide energy savings and investment costs out to 2035. Subsequently, by utilising UK Government forecasts for energy prices and the carbon intensity of electricity for various fuels (DECC 2011), we analyse total mitigation potential and net costs under different economic scenarios:

- Cost-effective: Measures are assessed using a private discount rate (5% real) and only those that repay their investment costs within their lifetime at this rate are deployed
- Cost-neutral: Measures are deployed such that between 2015 and 2035 total investments are matched by cost savings *in each sector* (implicit here is the assumption that savings from cost-effective measures could cross-subsidise cost-ineffective measures)
- Technical potential: All measures are deployed, irrespective of costs

### Consumption-based emissions

Finally, we estimate a time series of historical consumption-based (CB) emissions at the city-scale and project these forward to 2035. To compile the historical trajectory, we use *environmentally extended, multi*-*regional input*–*output* (EE-MRIO) analysis which uses monetary trade data to reallocate sectorial production emissions through global supply chains to the point of final consumption (Peters 2008). EE-MRIO analysis generates emissions intensities of consumption activities, also termed embodied emissions, represented as the carbon emitted (on average) per £million spent on a particular sector, as well as the geographical regions and sectors that these emissions originate within. We use EE-MRIO data developed by Lenzen et al. (2013) and applied to the UK (CCC 2013; Scott and Barrett 2015). In total 292 origins are considered: 110 sectors in the UK and 26 sectors in 7 global regions: *Europe, other OECD, China, India, developing Asia, Russia, rest of world*. Following Minx et al. (2013), we assume that the national-level sectoral carbon intensities in the tables are appropriate for the city-level, which is reasonable for the case given a relatively homogeneous country such as the UK. As the tables do not account for direct household emissions, due to fuels burnt in the home and in private vehicles, we add these sources to the CB account (directly from our PB baseline). Our method has many similarities with the *City Carbon Map concept* developed by Wiedmann et al. (2015), although our geographical disaggregation differs.^2^

The next stage of the analysis involves estimating Bristol-level final demand, in terms of money spent in each of these 292 sectors. This is comprised of government spending, capital investment, non-profit institutes serving households (NPISH) and household expenditure (which is dominant, accounting for two-thirds of the CB account; see SI). Again following Minx et al. (2013), we assume that national-level final demand for government spending, capital investment and NPISH can be downscaled on a simple (equal) per-capita basis for Bristol, as city-scale data are not available. To estimate household expenditure for Bristol, we draw upon the UK’s *Household Expenditure Surveys* (available from 2001 to 2013) and local demographic data from Bristol’s government censuses. By multiplying the vector of embodied emissions by the final demand vectors for each year, the historical CB trajectory is immediately obtained.^3^ Further details can again be found in the SI.

To make our projections, we use a simple *IPAT* identity (Nakicenovic et al. 2000) applied separately to Bristol’s final demand on UK and foreign products and carbon intensity terms:

$${\text{CO}}_{{2{\text{ - CB}}}} = {\text{ CO}}_{{2{\text{ - CB - UK}}}} + {\text{ CO}}_{{2{\text{ - CB - for}}}} = P \times \, \left( {{\text{FD}}_{\text{UK}} \times {\text{EI}}_{\text{UK}} + {\text{FD}}_{\text{for}} \times {\text{EI}}_{\text{for}} } \right)$$

where CO_2-CB_ are Bristol’s consumption-based emissions, *P* the population, FD the final demand per capita and EI the carbon intensity of spending (CO_2_e/£). The subscripts UK and *for* refer to expenditures on UK and foreign products, respectively. To project FD and EI forward, we simply use the average growth rates calculated from our historical data, with the population projection from government forecasts. Although this projection is relatively simple, it nonetheless closely resembles the UK-level forecasts reported recently in Scott and Barrett (2015) and CCC (2013), which use more complex methodologies that explicitly account for changes in the global productions systems consistent with a 4 °C warmer world. Thus, our CB emissions scenario lies midway between a global, business-as-usual economy and a fulfilment of the climate change commitments made at the Conference of Parties in Paris, 2015. In addition, we also report different forecasts that result from increasing or decreasing growth rates in final demand to reflect the influence of changing economic conditions.

## Results

### Production-based emissions estimates

**Fig. 1:**
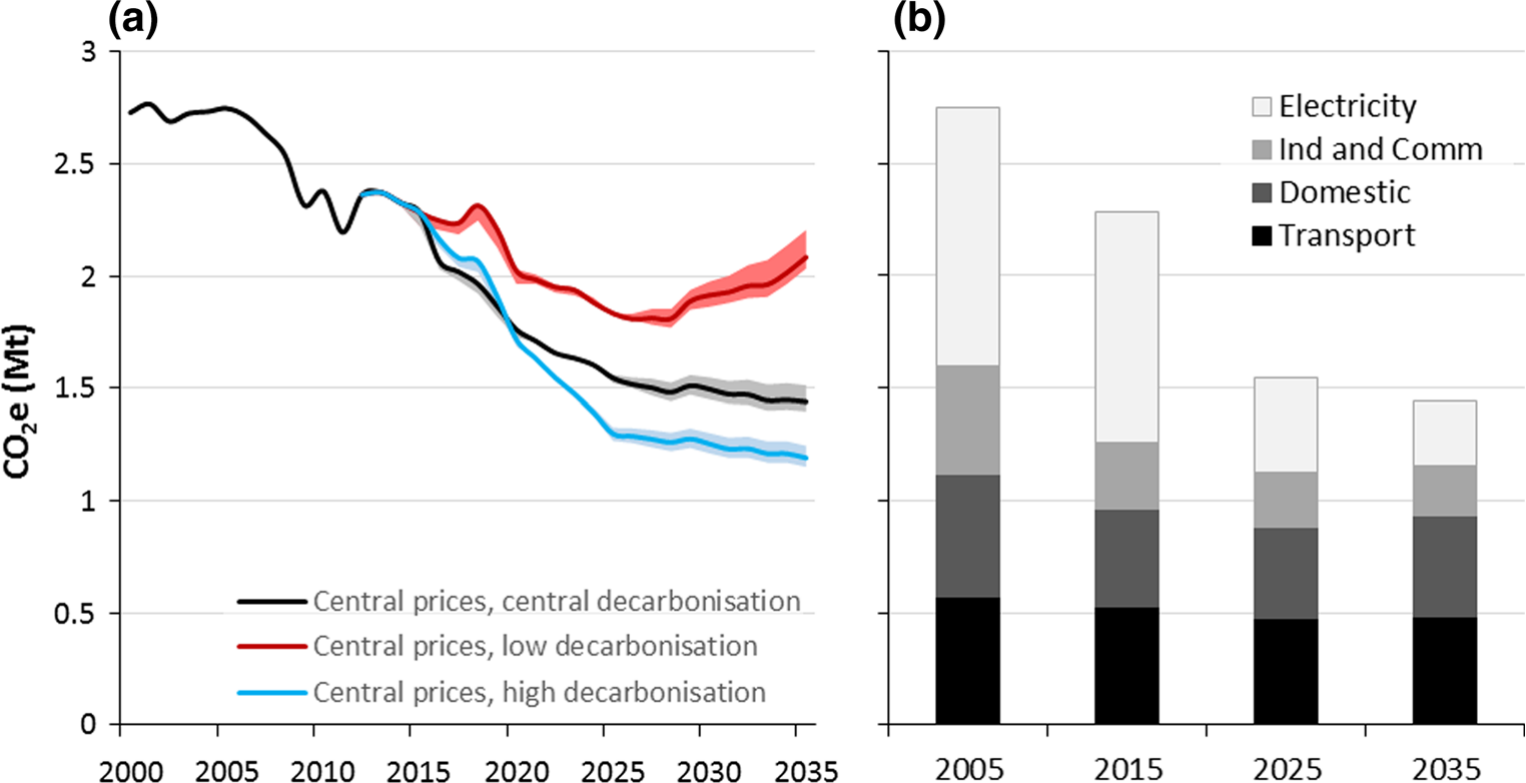
**a** Various baseline (BAU) projections for Bristol’s production-based GHG emissions. *Solid lines* indicate the trajectories for different grid decarbonisation scenarios and shaded regions show additional variations in due to high/low energy price forecasts from DECC (2011). **b** Emissions in the central prices, central decarbonisation scenario of **a** broken down by sector (‘Ind and Comm’ refers to the ‘Industrial and Commercial sector’)

Figure 1a below shows the historical trajectory of Bristol’s PB emissions and our projections under business-as-usual with varying levels of grid decarbonisation and changes in energy prices. It is clear that the different UK decarbonisation scenarios offered by DECC have a much more significant impact upon the emissions projections than changes in demand due to price effects. Also of importance is that emissions reductions plateau beyond 2025, or even rise in the case of slow UK electricity decarbonisation. Figure 1b offers some indication as to why this is the case: the vast majority of forecasted emissions reductions result from decarbonisation of UK electricity, but as significant decarbonisation has been achieved by 2025 in the central and high scenarios, the relatively limited decarbonisation that occurs beyond that will be increasingly offset and eventually even overwhelmed by ongoing increases in energy demand.

**Fig. 2:**
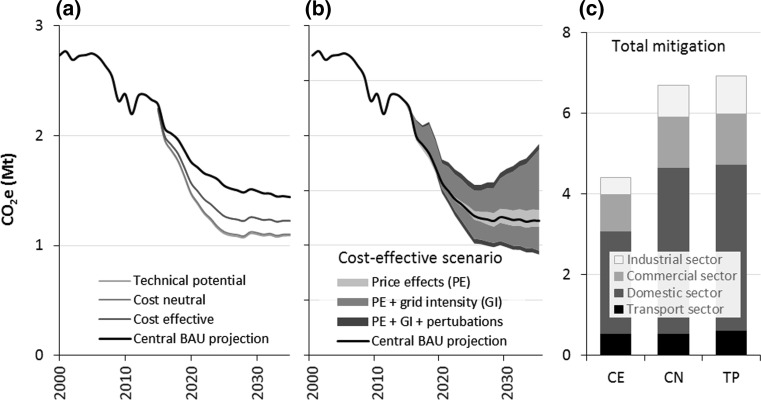
**a** Trajectories of Bristol’s GHG emissions for the three mitigation scenarios with central prices and decarbonisation shown alongside the BAU trajectory. **b** A sensitivity analysis of the cost-effective scenario indicating the differences in the projections with varying energy prices, grid carbon intensity and perturbations of model parameters (see SI). Variations are made additively, i.e. the full width of the shaded regions indicates the highest and lowest trajectories with different prices, decarbonisation and parameters varied simultaneously. **c** Cumulative emissions reductions from 2015 to 2035 in each scenario under central prices and decarbonisation

In Fig. 2, results from the cost-effective (*CE*), cost-neutral (*CN*) and technical potential (*TP*) mitigation scenarios are shown. Figure 2a shows the resulting three trajectories with central decarbonisation and energy price projections; Fig. 2b shows the sensitivity of the *CE* scenario to decarbonisation rates, energy prices, and perturbations of the most uncertain model parameters; and Fig. 2c shows the cumulative emissions reductions from 2015 to 2035, under central projections.

The CE, CN and TP trajectories reduce Bristol’s 2035 CO_2_e emissions by 55.2, 59.6 and 60.1% relative to 2000 levels, or by 15.1, 23.5 and 24.4% relative to the central BAU trajectory in 2035. In terms of cumulative mitigation and again relative to the central BAU forecast, the emissions reductions are 4.4, 6.7 and 6.9 Mt, respectively, with a dominant proportion of this achieved in the domestic sector. From 2015 to 2035, the three scenarios require investments of £1, £3 and £5 billion while generating cost savings of £3, £4.1 and £4.3 billion, respectively (in undiscounted terms). Therefore, while there is only a negligible difference between the CN and *TP* scenarios in terms of carbon and cost savings, there is a significant difference in investment requirements. This is predominantly due to public transport measures, which in our case have high costs and save only marginal amounts of carbon. However, there are two points to note here. First, the deployment of public transport measures is strongly motivated by many benefits other than saving energy and carbon, such as meeting air quality legislation and achieving social and economic benefits by reducing congestion. Second, the embodied emissions in vehicles and infrastructure become highly important when comparing the environmental impacts of public transport with private vehicles, such that from a lifecycle analysis perspective public transport measures have much greater carbon benefits than from a simple perspective of in-use emissions, as reflected in production-based carbon accounts.

The sensitivity test in Fig. 2b shows that—as expected—the CE trajectory would vary significantly with different trends in grid decarbonisation and energy prices, with the former again having the dominant influence. However, this test also shows that even a substantial perturbation of the most uncertain model parameters—namely the discount rate used to assess cost-effectiveness and the industrial and commercial deployment rates—adds very little additional uncertainly to the CE trajectory (see SI for more information).

### Consumption-based emissions

**Fig. 3:**
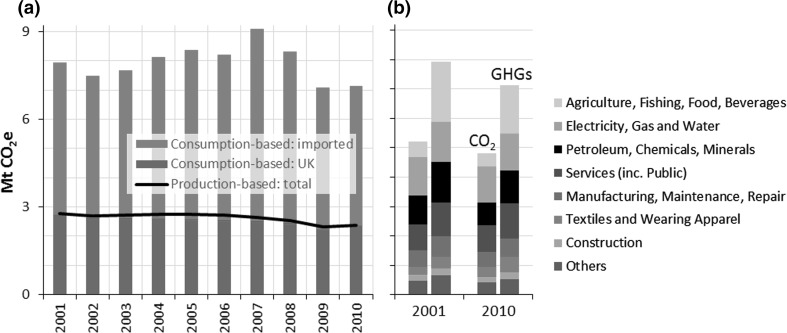
**a** Historical baseline for Bristol’s consumption-based GHG emissions disaggregated into those occurring in the UK and those imported. Production-based emissions over the same period are shown for comparison. **b** Consumption-based CO_2_ and GHG emissions in 2001 and 2010 disaggregated by sectors/product groups. ‘Electricity, gas and water’ here includes direct household emissions and ‘minerals’ refers to ‘non-metallic mineral products’

The historical time series of consumption-based (CB) emissions for Bristol are shown in Fig. 3, disaggregated in Fig. 3a into those emitted within UK territory and those emitted abroad and embodied in products destined for UK final consumption (imported),^4^ and in Fig. 3b by various sectors/product groups. Production-based emissions over the same period are displayed for comparison. Perhaps the most striking aspect of this figure is the discrepancy between the PB and CB trajectories. It is well known that CB emissions in developed countries with service-based economies tend to be higher than PB emissions, and the UK is one of the highest net importers of carbon, with 55% of the emissions embodied in UK consumption being reported in 2013 from the production of imports (DEFRA 2015). For Bristol residents, we have found a factor of three discrepancy (when considering all GHGs), which is particularly large relative to other studies (Peters et al. 2012; Kanemoto et al. 2014). A major reason for this is the emissions from *agriculture, fishing, food and beverages* in conjunction with our inclusion of all GHGs. Figure 3b shows that emissions from this product group are substantial and dominated by non-CO_2_ gases: they make up 25% of total CB greenhouse gas emissions, but only 10% of CB CO_2_. And for cities these products are almost entirely imported. Similar statements apply to the *Petroleum, Chemical and Non*-*Metallic Mineral* product group: embodied emissions are substantial, significantly higher in GHG than CO_2_ terms (although less so than *agriculture,* etc.), and almost exclusively imported into the city.

**Fig. 4:**
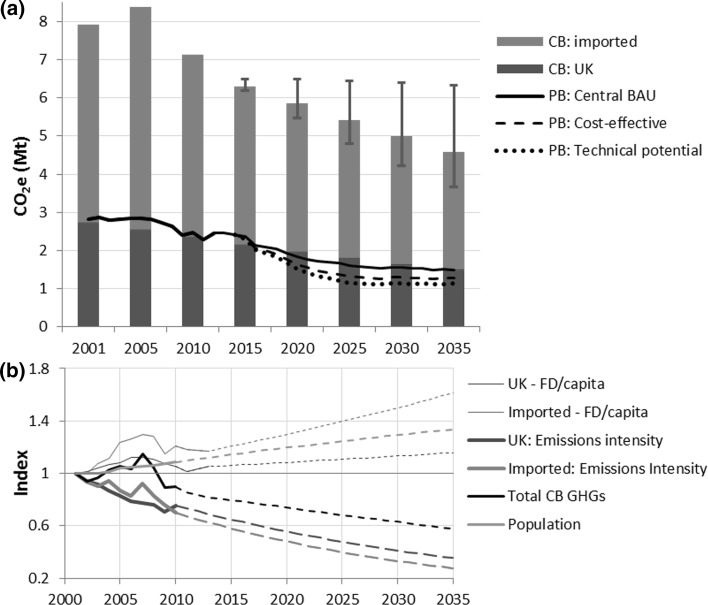
**a** Projections of Bristol’s consumption-based GHG emissions disaggregated into those occurring in the UK and those imported. Production-based emissions over the same period are shown. **b** Indexes of the IPAT terms used for the projection in **a**, with both historical (*solid lines*) and projected (*dashed lines*) data shown. FD refers to final demand and EI to emissions intensity

In Fig. 4a, our projections of Bristol’s CB emissions are shown, disaggregated by imports to the UK and alongside PB trajectories (both BAU and with mitigation). This suggests that CB emissions of Bristol may drop 40% by 2035 relative to 2001 levels. However, by then they are estimated to be still 3 times as large as the city’s PB emissions in the central BAU scenario. For comparison, Scott and Barrett (2015) forecast total UK CB emissions to fall steadily such that by 2035 they are 40–60% lower than 2000 levels depending upon whether international policies are consistent with a 4° or 2° warmer world. Thus, we could conjecture that even with a world successfully mitigating consistent with a 2° temperature rise, Bristol’s CB emissions would still be twice its PB emissions in 2035.

As noted previously, a full deployment of mitigation measures aimed at reducing Bristol’s PB emissions (i.e. the *TP* scenario) may reduce 2035 CO_2_e emissions by 24% relative to the central BAU trajectory in 2035. However, when these carbon savings are considered as a proportion of the projected CB emissions in 2035, the mitigation achieved is only 8%. Furthermore, this does not account for the carbon embodied in the mitigation measures deployed in the *TP* scenario, which would further reduce this 8%. For example, small-scale renewables may take 5–10 years—around a quarter to a third of their lifetimes—to mitigate their embodied emissions even when they are reasonably well sited (Bush et al. 2014). Thus, in the absence of broader changes in consumption patterns, extensive efforts to reduce the city’s PB emissions may have only a very minor impact upon the city’s CB carbon footprint.

In Fig. 4b, indexes of the IPAT terms used in the projection are shown. Given the historical variations in final demand and carbon intensity shown in Fig. 4b, it is clear that assuming single growth rates when projecting these parameters will not capture the full complexity of their dynamics. This is particularly significant for the final demand terms; however, the issue is mitigated by the additional temporal coverage of the household final demand data (2001–2013) relative to the carbon intensity data (2001–2010). Nonetheless, to test the sensitivity of our projections to this simplification, we shift the growth rates for final demand −1 and +1.5% relative to our central projections. This asymmetry reflects the intuition that our central projection is more likely to underestimate future demand due to the (arguably ongoing) financial crisis of 2007. The resulting variations in our predictions are indicated by the error bars in Fig. 4a. By 2035, it can be seen that the uncertainty in the CB projection is substantial, varying from 3.7 to 6.3 Mt around the central estimate of 4.6 Mt. However, the broad conclusions remain unchanged. Even with slow growth in final demand, projected CB emissions in 2035 are substantially higher than PB emissions. Conversely, under high growth, CB emissions still show reductions from 2010 to 2035.

**Fig. 5:**
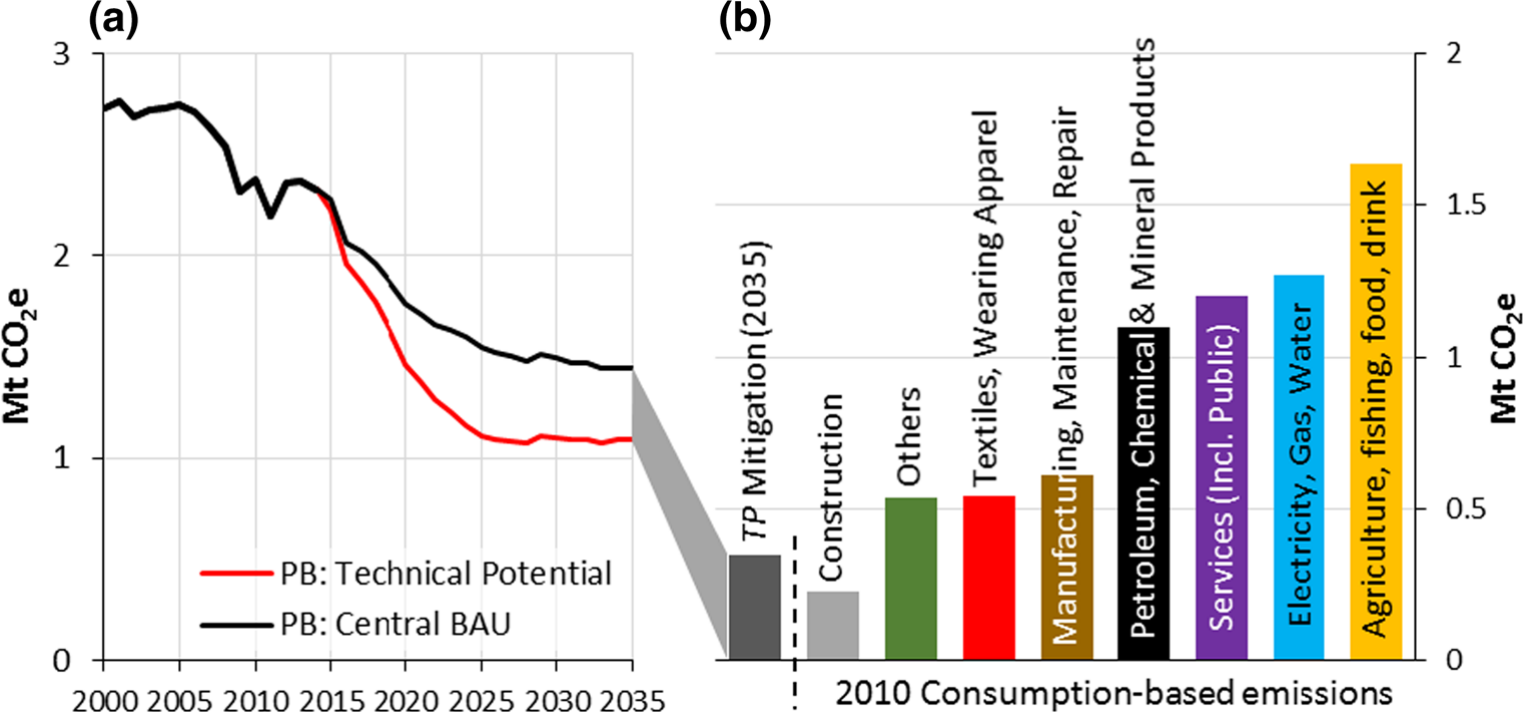
**a** Trajectories for Bristol’s production-based GHG emissions under BAU and the TP mitigation scenario, with central prices and decarbonisation. **b** Mitigation achieved in 2035 by the TP scenario shown alongside consumption-based GHG emissions for Bristol in 2010, disaggregated by various sectors. ‘Electricity, gas and water’ here includes direct household emissions. ‘Minerals’ refers to ‘non-metallic mineral products’

Figure 5 shows CB GHG emissions for Bristol in 2010, disaggregated by eight product groupings or sector groupings, alongside the BAU and *TP* mitigation trajectories for PB emissions. Although this is not a like-for-like comparison, as we are comparing 2010 CB emissions with forecasted 2035 PB mitigation, it is nonetheless instructive as the results show the magnitude of difference between projected technology savings from Bristol’s consumption-driven global impact. It can be seen immediately from Fig. 5 that all but one of the eight groupings (*construction*) was associated with significantly greater emissions in 2010 than the total annual mitigation projected for 2035 by the *TP* scenario. Perhaps most strikingly, 2010 CB emissions from the *agriculture, fishing, food and drink* sector grouping are nearly a factor of five greater than the total mitigation of the *TP* scenario in 2035. Emissions embodied in provision of *services* (*incl. public*) are three to four times higher than the 2035 *TP* scenario mitigation. And even CB emissions arising due to purchases of *textiles and wearing apparel* are significantly larger than the 2035 *TP* scenario mitigation.

## Discussion

We have described methods and frameworks for measuring and projecting the greenhouse gas emissions of cities and assessing mitigation options using both the commonly applied production-based approach and the rarely applied consumption-based approach. When applied to the city of Bristol, UK, our results suggest that GHG emissions may be three times larger from a consumption-based perspective relative to the production-based form of accounting. However, perhaps the most striking conclusion we find is the extent to which the emission reductions achieved by an ambitious programme directed at production-side mitigation are overshadowed by emissions associated with Bristol’s consumption.

This is not to say that such production-based mitigation should be disregarded. As we have demonstrated, more than half of the low-carbon measures that we consider may offer substantial carbon and cost savings, and the majority could be deployed at no net cost. Furthermore, there are various co-benefits that are increasingly well understood and now beginning to be incorporated into both government and privative decision-making processes (IEA 2014). These range from air quality improvements from efficient public transport systems to reductions in fuel poverty and increased resilience to energy price volatility from more efficient buildings (Jack and Kinney 2010; West et al. 2013). Such cost-effective measures should therefore be a top priority and utilising them could build the commitment and capacity needed to tackle less cost-effective options. However, our results post-2025 suggest the need for energy demand reduction to maintain ongoing decreases as decarbonisation is achieved. Thus, it is important to recognise that the way in which the deployment of low-carbon measures is governed will impact upon the mitigation actually realised in the longer term. Research indicates that the drivers called upon to motivate low-carbon action will shape their longer-term potential—with market-based appeals to individual self-interest likely to undermine citizen-based commitment to ongoing change, and top–down, technocratic styles of deployment likely to undermine rather than build the social capital and institutional learning needed for deeper transitions (Gouldson et al. 2015; also see Millward-Hopkins 2016 for a summary of the literature).

Moreover, it is clear that a focus upon production-based emissions alone presents rather limited mitigation options for cities. It is useful here to consider the local government targets for emissions reductions. Under Bristol’s current climate strategy (Minshull et al. 2015), the city is committed to future CO_2_ reductions of 50% by 2025 and 80% by 2050. These are ambitious targets and our analysis of mitigation pathways suggests that meeting these will require going beyond (currently) cost-effective options and achieving mitigation close to our cost-neutral and technical potential scenarios (see also Gouldson and Millward-Hopkins 2016). Alternatively, Bristol were to engage in certified offsetting schemes outside of the city, following the lead of cities in Australia (Chen et al. 2016a).^5^ Furthermore, the city is now considering increasing these targets such that by 2050 the city is carbon neutral on a production-basis.^6^ Effectively, therefore, our analysis suggest that meeting these more ambitious targets could require production-side mitigation that goes beyond what we currently consider to be technically feasible (Gouldson and Millward-Hopkins 2016). Of course new carbon reduction options could become available, and the economic case to support different options could change.

More broadly, the less that is achieved through demand reduction, the faster and greater energy supply will need to decarbonise. Thus, it seems essential to consider additional, consumption-based mitigation opportunities. As indicated in Fig. 5, the 2010 consumption-based emissions related to a number of high-level sectors—such as agriculture, food and drink; services; even clothing and textiles—are (far) greater than the *total* mitigation that could be achieved by an ambitious deployment of production-side measures in 2035 across all sectors. Shifting the focus of the city’s mitigation efforts towards a broader, consumption-based perspective would open up a range of emissions sources that are likely to be essential for achieving deep decarbonisation.

Of course there is a significant question about whether cities generally or city councils in particular have the capacity, awareness or commitment needed to address consumption-based emissions. Should they wish to do so through policy, then various options are available, from product and procurement standards and city and infrastructure planning, to economic measures to incentivise product longevity and a sharing economy (Afionis et al. 2016). Although a focus on consumption frequently leads to a focus on households, public and private sectors within a city are significant procurers of goods and services and thus have some influence over consumption-based emissions. In particular, the provision of infrastructure, such as new homes and transport networks, demands a high volume of carbon intensive resources, which can be reduced by improved building design, building standards, increased recycling, supply chain efficiency measures and adaptive reuse (Giesekam et al. 2014), and their effective planning and management can also shape user behaviours and thus broader consumption-based emissions.

More specifically, our analysis highlights the particular significance of the food and drink sector in shaping consumption-based emissions within Bristol. We estimate that emissions embodied in Bristol’s consumption of food and drink in 2010 are around five times the mitigation that could be achieved in 2035 if the city invested £3–5 billion to utilise all of the options associated with the cost-neutral or technical potential scenarios on the production side. If in Bristol, as within the UK more broadly, around 20% of all food is wasted (WRAP 2012), then the emissions embodied in the city’s food waste are of a similar magnitude to the mitigation that could be achieved through these ambitious scenarios in 2035.^7^ Intuitively, we expect the upfront costs of substantially reducing food waste to be much smaller than the billions of investment required by these scenarios. It has been demonstrated that reducing food waste, in both households and food-related sectors (e.g. hospitality), achieves cost savings for both businesses and households (WRAP 2014). Indeed, there are already initiatives such as *The Real Junk Food Project* (www.therealjunkfoodproject.org) that are attempting to address this issue via an innovative business model strongly rooted in both social and environmental outcomes, which aspires to reduce food waste (both at the household level and further up the business supply chain), thus moving towards a more circular economy, while simultaneously providing food affordable to those in financial difficulties. Providing sufficient policy and financial support for such civic initiatives to expand could be one step to reducing Bristol’s carbon footprint much more cost-effectively.

Although food waste is perhaps the lowest-hanging fruit of potential consumption-side mitigation strategies, another opportunity of particular relevance to Bristol—which could have an impact upon consumption-based emissions sources more broadly—is to expand the use of local currency.^8^ Such currencies have the potential to help relocalise consumption (Seyfang and Longhurst 2013), bringing more of Bristol’s carbon footprint into the scope of production-based accounts and potentially reducing carbon intensities of consumption (in cases where the intensities of local production are lower, or can be made lower, than for imported goods). However, the environmental benefits of localism are by no means inevitable and arguably are often exaggerated (Dittmer 2013). Furthermore, it is far from certain that bringing consumption-based emissions into the scope of production-based accounts would make them easier to address. Other more targeted measures that relate to the sharing economy, such as car pooling, tool sharing or swap shops, may be more certain to reduce carbon footprints. But their narrower focus would mean many such schemes would be needed to achieve significant mitigation, which may in turn be counteracted by the rebound effects that tend to arise under money-saving environmental interventions (Ottelin et al. 2015).

## Conclusions

The analysis presented in this paper suggests that it is imperative that consumption-based measures and mitigation options are more widely adopted and explored at the city-scale. The wider application of the methods we have developed is required if cities are to engage much more actively in consumption-based carbon mitigation. Although some researchers have explored consumption-based mitigation options relating, for example, to increasing product lifetimes and alternative business models (Barrett and Scott 2012), there is a need for more detailed options appraisal at the city-scale if consumption-based emissions are to be significantly reduced. This could be facilitated if the many organisations that are developing frameworks to encourage cities and communities to adopt low-carbon plans extended the boundaries of their work to consider not only production but also consumption-based emissions.

However, we add three important caveats to this call for greater emphasis on consumption-based carbon accounting in cities. First, by highlighting the potential for consumption-based carbon management, we stress that we do not seek to challenge or undermine the critical importance of production-based mitigation in cities. Ambitious action is needed on all forms of carbon mitigation, and many production-side measures in cities are highly carbon and cost-effective. If their deployment is governed carefully, then the financial benefits could help to build the capacities needed to tackle less cost-effective options. But from a climate change perspective, it is clear that a focus upon production-based emissions alone presents rather limited mitigation options.

Second, we recognise that the institutional capacities, policy instruments or governance interventions that have been developed to support production-based mitigation may be different from those needed to support unusual or innovative consumption-based mitigation such as minimising food waste. We also acknowledge that the institutional capacities needed to address consumption-based carbon emissions tend to be under-developed at all levels and that they are often entirely absent at the city-scale. Some of the new environmental policy instruments that have been developed by national governments in recent years could be adapted and applied to consumption-based emissions at the city-scale. But given the limited capacities of many city-level governments, it seems likely that new approaches that rely less on traditional forms of government and more on new forms of governance driven not only by government but also by a wider range of public, private and civic actors will be needed. Innovative initiatives relating to the circular or sharing economy or parallel currencies exemplify the potential of new forms of governance.

Finally, we recognise that there are likely to be difficult social, cultural and political barriers to overcome in the pursuit of carbon mitigation through consumption-based approaches. Within a growth-dependent economy, calls from majority seeking politicians for citizens to help to address climate change by reducing their consumption are perhaps unlikely. Indeed, many politicians frequently advocate the precise opposite. And the reality of rebound effects means that reductions in consumption of one product may simply lead to increases in consumption elsewhere (Druckman et al. 2011). Such contradictions remain a core challenge to both production- and consumption-side mitigation strategies, but they are perhaps most consequential for the latter. Our discussion therefore links the importance of city-scale measurement and mitigation of consumption-based emissions into much wider and deeper debates about the desirability of economic growth and the impacts of, and alternatives to, a materialistic consumer society.

## Electronic supplementary material

Below is the link to the electronic supplementary material.

Supplementary material 1 (DOCX 342 kb)

## Footnotes

1 The sectors we consider incorporate all those included in the GPC standard aside from the waste sector. However, in Bristol this accounts for <5% of the city’s emissions (see Bristol’s Environmental Statement 2014/15; www.bristol.gov.uk/policies-plans-strategies/).

2 Specifically, Wiedmann et al. (2015) disaggregate consumption-based emissions into those occurring within the city, regionally, nationally and internationally, while we only disaggregate into national and international.

3 Our estimate of 2004 per-capita CB emissions for CO_2_ only is close to that of Minx et al. (2013); 13.9 versus 12.2 t, respectively, who use different data sets to derive final demand.

4 The difference between our UK CB proportion and PB account for Bristol is negligible in Fig. 3, as the UK proportion includes emissions embodied in products consumed in Bristol but produced elsewhere in the UK. Ideally, we would split our CB account into a ‘domestic’ (Bristol) proportion, ‘UK’ (outside of Bristol) proportion, and an imported (international) proportion, but unfortunately available data determine that we combine the first and second of these into a single estimate.

5 As Chen et al. (2016a, b) point out, investing in offsetting schemes elsewhere may also reduce the consumption-based footprint of the region making the investment, if trade linkages between the regions are significant.

6 See http://news.bristol.gov.uk/bristol-increases-its-ambition-and-aims-to-be-carbon–neutral-by-2050 (accessed 24/10/2016).

7 See our SI for a back-of-the-envelope calculation that increases our confidence in this assertion.

8 The *Bristol Pound* was the first city-wide currency in the UK and the first to be accepted to pay taxes. The mayor also announced that he would take his full salary in the local currency.
