# Supplementary material for: Uncovering blind spots in urban carbon management: the role of consumption-based carbon accounting in Bristol, UK
Source: Reg Environ Change. 2017 Feb 3;17(5):1467–78. doi: 10.1007/s10113-017-1112-x (PMC6991983; doi:10.1007/s10113-017-1112-x)
Supplement: Supplementary file 1 — Supplementary material 1 (DOCX 342 kb) [file 10113_2017_1112_MOESM1_ESM.docx]

**Supplementary Materials: Further details on methods**

**Uncovering Blind Spots in Urban Carbon Management:**

**The Role of Consumption-Based Carbon Accounting in Bristol, UK**

*Joel Millward-Hopkins, Andrew Gouldson, Kate Scott, John Barrett and Andrew Sudmant*

*Sustainability Research Institute, University of Leeds, UK*

*In these supplementary materials we expand upon the methodology outlined in our paper in order to fully describe our data sources, assumptions and calculation processes. We also present some further results for the more interested reader.*

# Production-Based Emissions: BAU

In the first stage of the method we develop a baseline, business-as-usual (BAU) trajectory for city-scale production-based (PB) emissions, i.e. the carbon emitted either directly within the city’s boundaries or indirectly via electricity use. We focus upon all greenhouse gases, measured as CO_2_e.

Our starting point is historical city-scale emissions data. To develop a BAU trajectory, we project these data forward by utilising city-level population forecasts and national-level emissions scenarios. For our case in this paper, Bristol in the UK, all these data are freely available through the government’s open data site (<https://data.gov.uk>):

- Local authority (LA) level emissions data disaggregated into *domestic*, *industrial and commercial*, and *transport* sectors and various subsectors is available from The Department for Energy and Climate Change (DECC; CO_2_ only from 2005-2012)
- Both UK- and city-level population projections are regularly updated by the Office for National Statistics (ONS; currently to 2037)
- UK-level projections of emissions and the carbon intensity of electricity supply are also available from DECC (both CO_2_ and all GHGs out to 2035; disaggregated by nine sectors). These are available for various scenarios with differing energy prices, decarbonisation paths, and policies

To make our projections, we first match the national-level emitting sectors to the city-level sectors, aggregating into clusters where necessary (as shown in table 1). We then convert the local CO_2_ emissions to all GHGs by using the ratios of CO_2_e to CO_2_ for each national-level sector/cluster. Third, we calculate growth rates in *per-capita* emissions for these national-level sectors/clusters. Using these growth rates, we then take the latest (2012) city-level, per-capita emissions for each sector/cluster and project these forward to 2035. We therefore assume that the per-capita growth rates in emissions at the city- and national-levels are equal for each sector/cluster. Finally, we aggregate these projections into total emissions using the city’s population projections. For the case, we utilise the various UK-level emissions projections to compile a number of baselines for Bristol relating to nine permutations of central/low/high prices and central/limited/high decarbonisation.

|  | **National-level** | | **City-level** | |
| --- | --- | --- | --- | --- |
|  | **Disaggregation** | **Time frame** | **Disaggregation** | **Time frame** |
| **Emitting sector** | Agriculture Industrial processes Waste management Business Public | 1990-2035 | Ind' & Com' *(other fuels)* | 2005-2012 |
|  | Energy supply |  | Ind' & Com' (electricity) Domestic (electricity) |  |
|  | Residential |  | Domestic (other fuels) |  |
|  | Transport |  | Transport |  |
|  | LULUCF |  | LULUCF |  |

*Table 1: National-level sectors from the DECC emissions scenarios matched to the city-level, local authority emissions sectors (aggregating where necessary, as indicated by the shading). Note that for the case, Bristol emissions from Land Use and Land Use Change and Forestry (LULUCF) are negligible, at less than 0.3% of total city-level emissions*

# Production-Based Emissions: Mitigation Scenarios

## Overview

We then explore city-level mitigation scenarios for PB emissions across the domestic, commercial, industrial and transport sectors. As described below, for each sector we (i) identify a range of applicable measures, (ii) assess their per-unit investment costs and energy savings, and (iii) estimate their city-wide deployment potentials. Throughout this process we consult with local partners to ensure the lists of measures are appropriate, costs and savings reasonable, and deployment levels realistic. We then assess the total city-level mitigation that could be achieved across these sectors under different scenarios, by utilising national-level carbon intensities (CO_2_/kWh) and prices of energy (£/kWh).

Each sector has a different unit of analysis, namely single house (domestic sector), unit floor-space (commercial sector), unit energy saved (industrial sector), and passenger-km provided and single vehicle (public and private transport sectors). Thus for the commercial sector, costs and savings for a measure relate to one m^2^ of floor-space, and the deployment potential is the number of m^2^ of floor-space viable for the measure throughout the city. Much of the cost and savings data we use is applicable throughout the UK, as are the methods we use to estimate city-level deployment potentials. Public transport is the main exception to these generalisations, being reliant upon extensive locally-specific considerations, which involves us consulting with local experts and transport planners.

## Calculating Annual Carbon Savings

Methodologies for estimating annual carbon savings in the domestic and commercial sectors are outlined in figures 1 and 2. Annual carbon savings per-unit of each measure are simply multiplied by the number of units deployed in the mitigation scenario (houses or m^2^ of floor-space). Per-unit carbon savings are obtained from the energy savings data we describe below and the associated emissions intensities. We also account for the interactions that occur when multiple measures are deployed within the same building, which can reduce the savings achieved in the case of, for example, solar photovoltaics and efficient lighting.

Calculating annual mitigation in the industrial sector frequently requires a different approach due to differences in the data available (see figure 3) and this was apparent in the case study city. First, using the method described below, we estimate total, city-level industrial consumption of electricity, gas and coal and hence the energy use for which each measure may be applicable (i.e. the deployment potentials in GJ). Here we also account for expected deployment of the measures under BAU. We then estimate the energy savings achieved by each measure by multiplying the deployment potential (GJ) by the measure’s efficiency improvement (%). Carbon savings are then easily obtained by considering the relevant national-level emissions intensities.

Mitigation in the transport sector is often significantly more difficult to calculate and again this was evident in the case study city. Conceptually, as shown in figure 6, the process is relatively simple. It involves compiling emissions intensities for each mode of transport (CO_2_e/pkm) and city-level mode share (pkms) out to 2035. Total emissions for the mitigation scenario are obtained by multiplying these data together. Mitigation is simply the difference between this and the BAU trajectory. However, the emissions intensities in particular are highly complex to derive.


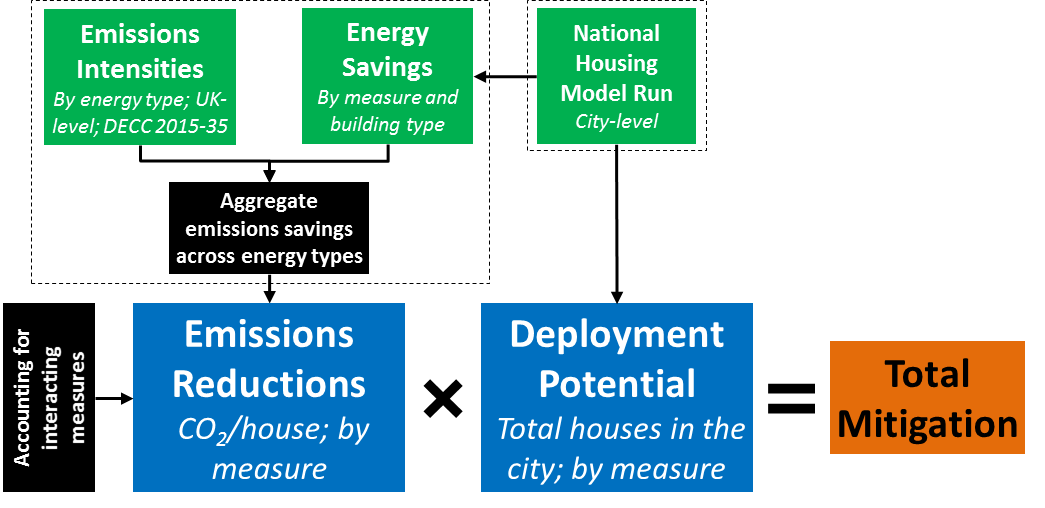


*Figure 1: Flowchart outlining the domestic sector methodology*


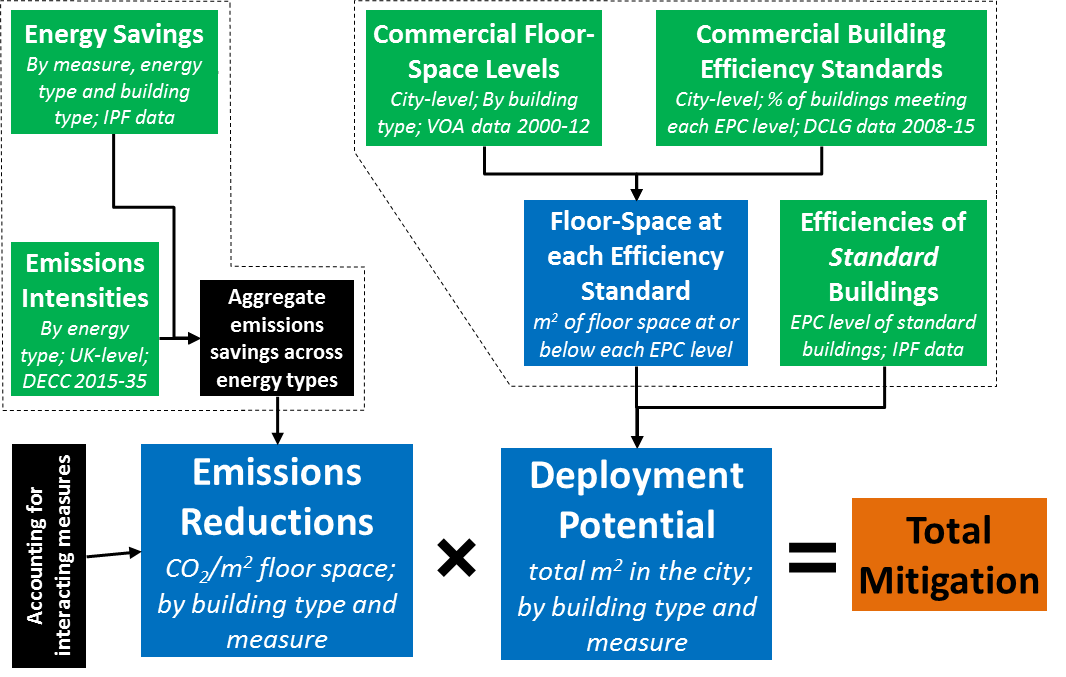


*Figure 2: Flowchart outlining the commercial sector methodology*


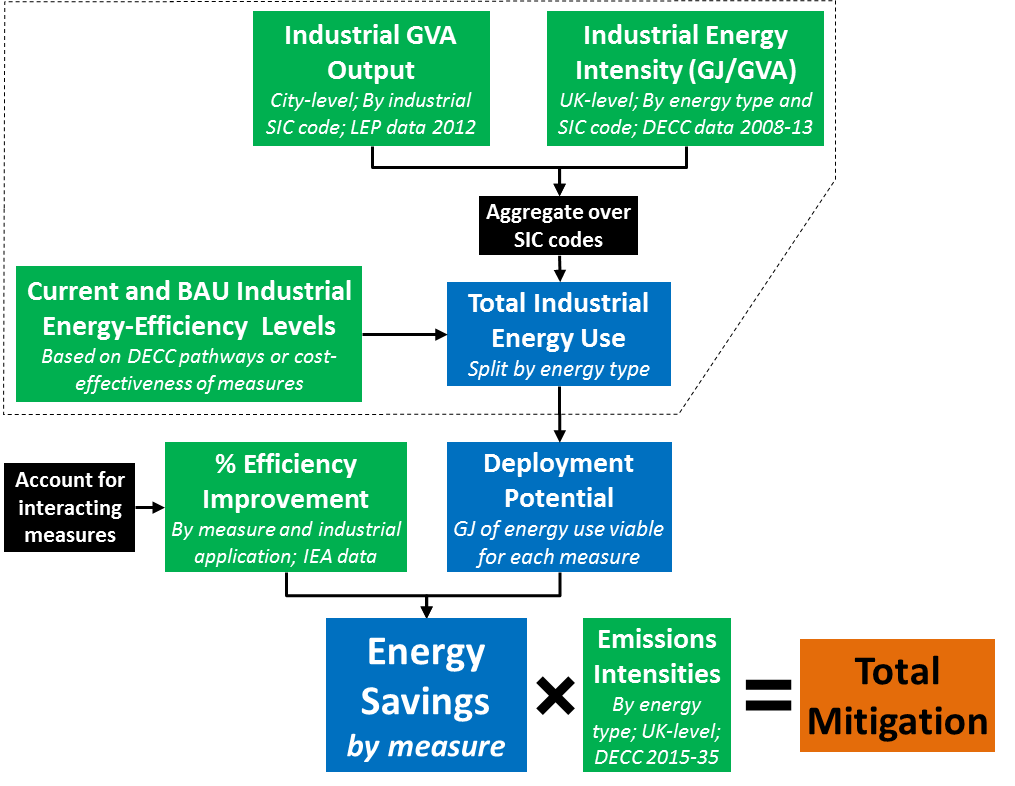


*Figure 3: Flowchart outlining the industrial sector methodology*

For our case study, to estimate Bristol residents’ travel activity (in pkms/capita by mode; figure 4), we use a combination of city- and national-level data. Bristol-level data was only available for commuting travel, thus we supplement this with national-level data for relating to other purposes (business, leisure, shopping etc.). The former data are obtained from local censuses^[[1]](#footnote-1)^ (2001-2011) and the latter from the National Travel Surveys^[[2]](#footnote-2)^ (2002-2013). Travel activity shows a consistent, steady decline over the 10-12 years for which we have data and hence to estimate future travel activity we simply use liner regression applied to the pkms/capita for each mode of travel. For consistency, we ensure that pkms/capita aggregated over all modes is the same in both BAU and mitigation scenarios. Therefore, the public transport measures we consider in the case only induce a *mode-shift* but *not a demand reduction*.

To estimate emissions intensities for the case study city, we begin with the standard, current UK-level emissions intensities (CO_2_e/vkm) for local buses, coaches, local rail, and cars/vans (petrol and diesel; small, medium and large) used by DEFRA^[[3]](#footnote-3)^. We combine these with National Travel Survey data of average occupancy levels to convert from emissions per vkm to emissions per pkm. We then project BAU reductions in emissions intensity out to 2035 using the efficiency improvements forecast for different vehicle types in the Department for Transport’s *Road Transport Forecasts* *2013*^[[4]](#footnote-4)^ and, for electric vehicles, by the IPCC^[[5]](#footnote-5)^. For cars/vans, we then aggregate the data for different vehicle-types into average emissions intensities by considering (i) the proportion of activity of each type of vehicle at the UK-level using the projections of the *National Atmospheric Emissions Inventory*^[[6]](#footnote-6)^ (NAEI) and (ii) the additional hybrid vehicles deployed in the mitigation scenario, as described in the following section.

← data projections →

*Figure 4: Assumed activity levels per-capita in Bristol for both the BAU and mitigation scenarios measures in 1000s of pkm’s per year and split by mode*


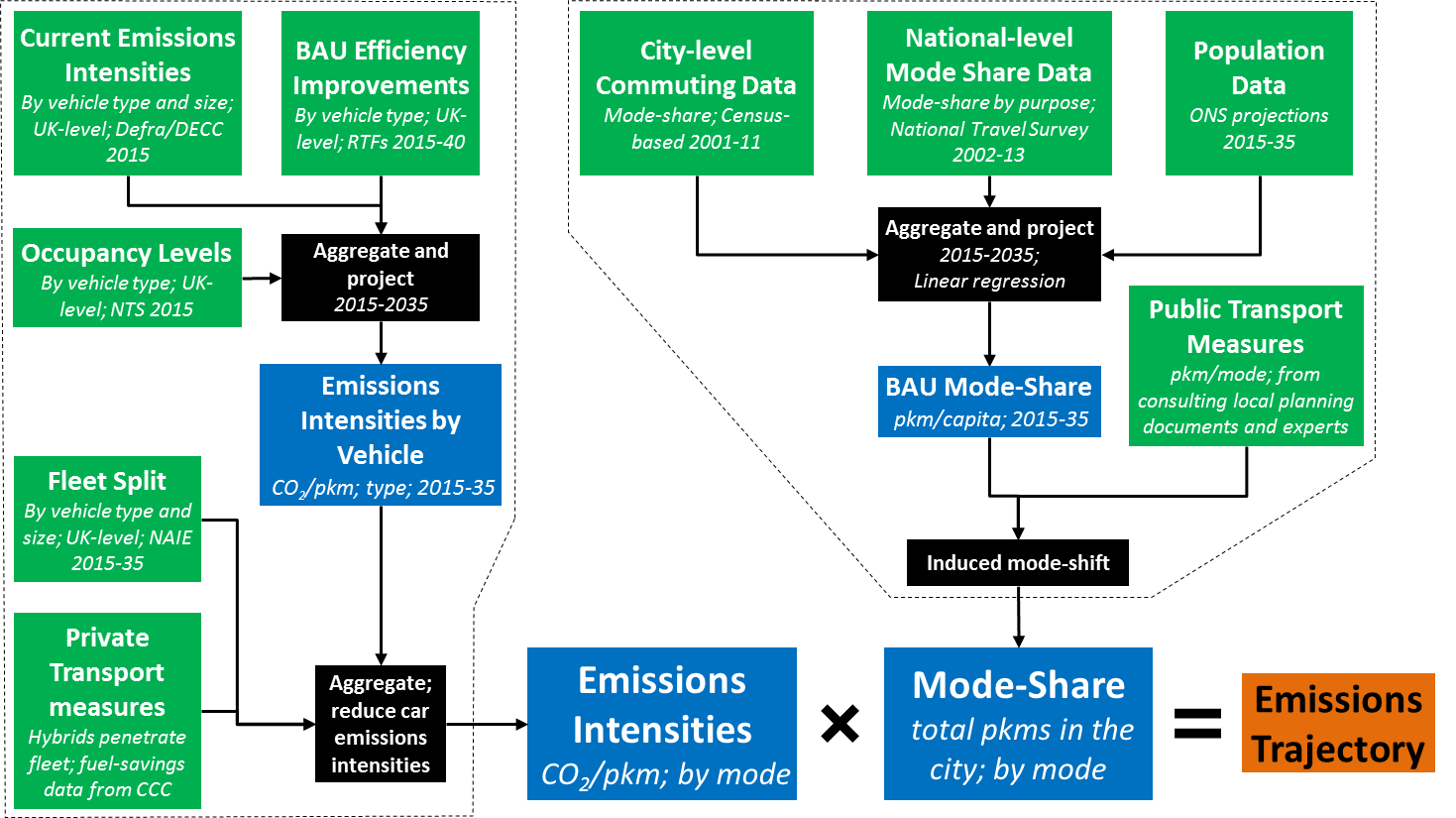


*Figure 5: Flowchart outlining the transport sector methodology*

## Sectoral Input Data

### Domestic Sector

For the domestic or residential sector in the case study city, the list of measures, their lifetimes, and their costs and energy savings (electricity, gas, and other fuels) are outputs from the UK’s National Housing Model (NHM), which was developed by the *Centre for Sustainable Energy* as commissioned by DECC^[[7]](#footnote-7)^. It contains a detailed representation of the full English housing stock including information upon currently installed building fabric, insulation levels, heating systems, etc. at the level of individual properties. This allows the model to assess what measures are appropriate for a particular city’s domestic sector, how many houses each measure would be suitable for, and what energy savings would be expected assuming the household maintains the same heating regime post-installation of each measure. Due to this high level of detail we consider the input data for this sector the most robust of the four.

### Commercial Sector

For the commercial sector in the case study city, we obtain lists of measures and their lifetimes, costs, and energy savings (electricity and gas) from the review of the Investment Property Forum^[[8]](#footnote-8)^ (IPF), which are considered to be appropriate throughout the UK. Measures are grouped into different building types, namely *offices*, *retail properties*, and *warehouses*. The (marginal) costs and energy savings provided by the IPF are relative to a standard *market refurbishment*. Data are also supplied by the IPF describing the current Energy Performance Certificates (EPCs) of these standard buildings.

To calculate city-level deployment potentials we utilise LA-level data describing:

- Existing commercial floor-space by building type: i.e. by *offices*, *warehouses* and *retail* buildings from the Valuation Office Agency^[[9]](#footnote-9)^ (VOA)
- The distribution of EPCs reported for the current (2015) commercial building stock, from the Department for Communities and Local Government^[[10]](#footnote-10)^ (DCLG).

We use these data together to estimate the floor-space in Bristol currently meeting each EPC level. Then, by considering this in conjunction with the EPCs of standard *office*, *retail*, and *warehouse* buildings offered by the IPF, we estimate the areas of floor-space currently at or below *market refurbishment* levels for each building-type. We consider these floor-space areas to be eligible for further measures. Implicitly, therefore, we assume that under BAU only *market refurbishments* take place and the area of commercial floor-space in Bristol remains static. This appears reasonable as for the periods within which data are available there only negligible changes in the distributions of EPCs of commercial buildings in Bristol (from 2008-15) and the existing commercial floor-space (2000-12).

An example is instructive here: The IPF suggest that *retail* buildings that have undergone a *market refurbishment* achieve an EPC level *C*, while 90% of the EPCs reported by Bristol’s commercial building sector in 2015 were level *C* or lower. Therefore, in this case we would assume that 90% of the (1.1 million m^2^) *retail* floor-space in Bristol is at or below market refurbishment level and is therefore viable for measures.

### Industrial Sector

For industry in the case study city, we use measure specific data from the International Energy Agency’s World Energy Investment Outlook^[[11]](#footnote-11)^. We consider only cross-cutting industrial measures^[[12]](#footnote-12)^ that are grouped into efficiency improvements to *boilers/steam systems*, *furnaces/process heaters*, *refrigeration*, and *motor driven equipment*. This final category is further split into *pumps, fans and compressed air systems*. The IEA data suggests the percentage improvements in efficiency that can be achieved by each measure, alongside the investment requirements per-unit of energy saved. Deployment potentials for each measure are difficult to evaluate accurately due to issues of confidentiality around industrial operations and hence we make a number of assumptions. Our approach involves three main stages:

1. We estimate total, city-level industrial consumption of electricity, gas and coal
2. We assume that all gas is used in *boilers/steam systems*, coal in *furnaces/process heaters*, and electricity in *refrigeration* and *motor-driven equipment*
3. We estimate current and BAU deployment of each of the IEA measures and hence the proportion of fuel use that is viable for each measure in the mitigation scenarios

To estimate (1) for the case study, we use Bristol-level data describing the output (in GVA) of each industrial sector specified by its (2-digit) Standard Industrial Classification (SIC; provided by the West of England Local Economic Partnership^[[13]](#footnote-13)^), in conjunction with UK-level data describing the energy use per unit GVA of these SIC sectors (from the ONS environmental accounts^[[14]](#footnote-14)^) which we split by fuel-type using DECC industrial energy use data.

To estimate (3) for the case study, we use the pathways outlined in the DECC *Industrial Decarbonisation and Energy Efficiency Roadmaps to 2050^[[15]](#footnote-15)^* and where this is not possible we make some simple assumptions. These pathways describe current and potential deployment of industrial efficiency measures out to 2050 in 5 year intervals in terms of the (increasing) percentages of UK industry for which each measure is deployed. However, only about one third of the IEA measures match with a measure listed in these pathways. In these matching cases, we cut down the deployment potentials of the measures by the percentages they are forecast to be deployed by in 2035 in these pathways. For the remaining measures, we use a simple rule: We assume that highly cost-effective measures will be deployed to 2/3^rds^ of their total potential under BAU (i.e. those that are cost-effective at a 5% discount rate), that moderately cost-effective measures will be deployed to 1/3^rd^ of their potential (i.e. those that are cost-effective at a 3 to 5% discount rate), and those that are cost-ineffective will not be deployed at all without concerted mitigation efforts (i.e. those that are not cost-effective at a 3% discount rate).

As these calculations represent some of the more uncertain in our model, we test the sensitivity of the results to a large perturbation in our assumptions as described below.

### Transport Sector

#### Public Transport

For the public transport measures in the case study city, our preliminary and provisional lists draw on data from the previous mini stern review of the Birmingham City Region^[[16]](#footnote-16)^. This data provides either unit-costs per for a particular measure and the mobility per unit typically provided (e.g., costs per km of cycle lane and average occupancy) or costs for a full project (e.g. a bus rapid transport network) and the full mobility provided in pkms per year. After reviewing regional transport plans, we scale these costs and mobility figures to better represent the Bristol context. We then check these figures by consulting with local government and transport planning experts to determine if the list of measures is appropriate and the costs and deployment levels realistic, thus we refine our estimates based upon this feedback. As for the industrial sector, there are potentially significant uncertainties here. However, these have a negligible impact upon the mitigation trajectories, as the transport sector’s city-level mitigation contribution is small. Note that our assumptions do have a significant impact upon the economics of the transport sector, but in this paper we are primarily concerned with emissions.

#### Private Transport

All the private transport measures considered for application in the case study are hybrid vehicles. To evaluate their potential, we again begin with data from Birmingham City Region, which in this case was gathered from consultation with the UK Committee of Climate Change. This data describes incremental costs of hybrids and the associated fuel use per km. Four types of hybrid are considered – micro, mild, full and plug-in – and these are disaggregated into small, medium, and large vehicles and then again into diesel and petrol vehicles.

To estimate how many of each vehicle could be deployed in the city, above and beyond BAU, we first estimate the current, city-level vehicle stock by considering:

1. The number of cars per capita existing in Bristol from the local census (0.43 in 2001 and 0.45 in 2011)
2. National-level data describing the split of this fleet between small/medium/large and petrol/diesel/electric cars, from the *NAEI^[[17]](#footnote-17)^* and the *Road Transport Forecasts 2011^[[18]](#footnote-18)^*

In the BAU and mitigation scenarios, we assume the number of cars per capita in the city remains unchanged from current levels and that hybrid vehicles are deployed at a fixed penetration rate each year. For BAU this rate set to 0.25% per year such that 5.3% of vkms are powered by electricity by 2030, as projected in the *Road Transport Forecasts 2013*. In the mitigation scenario, the rate is increased to 2.8% such that the fleet is 100% hybrid vehicles by 2050, which is the target consistently stated by the CCC in their progress reports^[[19]](#footnote-19)^. The fuel use per vkm data for the hybrid vehicles allow us to estimate the reduction in the average emissions intensity of cars/vans under the mitigation scenario.

## Scenarios and Sensitivity Analysis

By integrating these investment-costs, fuel-savings, and deployment-potentials data with projections of energy prices and emissions intensities, we estimate the cumulative, city-wide mitigation achieved above-and-beyond business-as-usual out to 2035. We execute this for the three scenarios outlined in the main paper (*cost-effective*, *cost-neutral* and *realistic potential*). In each scenario, the annual deployment rate of each measure is set to 10% of its total potential and once its lifetime is exceeded it is immediately redeployed. Tables for each sector, including costs and carbon savings for every measure analysed, are included in Gouldson and Millward-Hopkins^[[20]](#footnote-20)^ (2015).

In the main paper we also report a sensitivity analysis in which we vary a number of parameters, including decarbonisation rates, energy prices, and perturbations of the most uncertain model assumptions. More specifically, the variation in our sensitivity tests is achieved via the variations outlined in table 2 below.

|  | | **Low mitigation** | **Central mitigation** | **High mitigation** |
| --- | --- | --- | --- | --- |
| **Decarbonisation:** from DECC grid intensity projections *(CO_2_e/kWh)* | | *no policy* | *central projection* | *cost-effective path* |
| **Prices effects:** from DECC Energy and Emissions Projections 2014^[[21]](#footnote-21)^ | | *low prices* | *central prices* | *high prices* |
| **Deployment potentials** | Commercial sector | Deployment potentials halved | Based on current EPC levels of Bristol’s commercial buildings | Deployment potentials doubled**^i^** |
|  | Industrial sector | Highly cost-effective measures: *BAU* deployment is **75%** of total potential  Moderately cost-effective measures: *BAU* deployment is **50%** of total potential | *ditto left* but **67%** of total potential  *ditto left* but **33%** of total potential | *ditto* *left* but **25%** of total potential  *ditto* *left* but **0%** of total potential |

**^i^** Unless this would exceed the total commercial floor space in Bristol

*Table 2: Parameter and modelling assumptions made in the sensitivity analysis*

# Consumption-Based Emissions

## Overview

Finally, we estimate a time series of historical, city-scale consumption-based emissions, projecting these forward to 2035. For this, we use data derived from *environmentally extended, multi-region input-output analysis* (EE-MRIOA). These methods have been described in detail in other work^21-25^, and hence here we offer only a brief overview.

EE-MRIOA can evaluate the emission impacts embodied in goods and services traded between nations and is recognised as the most appropriate tool to estimate consumption-based emissions accounts at the national and supra-national level^[[22]](#footnote-22),^^[[23]](#footnote-23),^^[[24]](#footnote-24)^. EE-MRIOA reallocates production emissions, which are point source emissions from sectors within a country’s territory, to the destination country of the final consumer through complex international trade flows^[[25]](#footnote-25)^.

Using input-output (IO) analysis, consumption emissions (F) are given by $F=f_{x}Ly$, where *f_x_* is the direct carbon intensity of production sectors, *L* is the effect of trade transactions (known as the Leontief Inverse), and *y* is the volume and composition of final consumption, i.e. the *final demand*. Carbon intensities for production sectors (*f_x_*) are calculated by dividing direct sector emissions (*f*) by the sector’s economic output (*X*). The Leontief inverse (*L*) calculates the ratio of upstream requirements (i.e. goods and services) to produce each sectors’ finished products. When multiplied by the vector of carbon intensities it provides carbon intensities for final products which includes the direct and indirect emissions produced along product supply chains to the point of purchase, referred to as total carbon intensities. Multiplying the total carbon intensities for domestic (and imported) products by a region’s final demand for domestic (and imported) products determines the emissions released globally in the production of goods and services consumed in that region – its consumption-based emissions account.

## Data sources

### Carbon Intensities

For our work, the IO model used to develop the total carbon intensities for products consumed in the UK was Eora, an EE-MRIO model developed by the Integrated Sustainability Analysis (ISA) group at the University of Sydney^[[26]](#footnote-26),^^[[27]](#footnote-27)^. This has been applied to analyse UK consumption-based emissions by other researchers^[[28]](#footnote-28),^^[[29]](#footnote-29)^. We use these same UK-level carbon intensities at the city-scale for Bristol, which is a reasonable simplification for a relatively homogeneous country such as the UK.

Eora provides a global transactions matrix showing inter-industry trade between 187 countries, which is inverted to produce the *L* matrix, for a time series from 1990-2010^[[30]](#footnote-30)^. Information on domestic intermediate sales and purchases are collected from the national economic accounts of each country where available. Alongside the domestic tables, countries report import tables. For each sector, the import tables report spend on imported products but the region of origin is not reported. Eora estimates trade between regions by disaggregating the imports matrix to show share by region using international trade data from United Nations commodity trade statistics database COMTRADE. Some estimation is required in the allocation of trade data to the sector classification used by different regions, and where countries do not report to COMTRADE, a proxy nation’s trade structures are used instead. Sales to final consumers, made up of households, government, capital and not-for-profit institutes serving households, are also recorded (*y* in the IO equation).

The level of sector resolution varies across countries, ranging from 511 sectors to 26 sectors. Using the common bottom denominator, a 26 sector harmonised system was also developed to enable easier cross-country comparisons and sector analysis. For the 98 countries where IO tables are not available, but total sectorial economic outputs are, a representative economy is applied using the average of tables from U.S., Japan and Australia. For missing years, a country’s IO table from a previous year is updated using available economic indicators. Global sectoral carbon dioxide emissions in Eora are sourced from a combination of data from EDGAR, UNFCCC and CDIAC.

### Final Demand: Downscaling to the City-Scale

When downscaling the UK-level (Eora) model to the city-scale the most important consideration is household final demand (HH-FD). As show in figure 6, this is by far the largest source of emissions with respect to other sources of final demand, accounting for 70% of 2010 CB emissions. To estimate HH-FD at the city level, we combine: (i) data from UK household expenditure surveys^[[31]](#footnote-31)^ to build a picture of the (UK average) spending profile of persons of various *economic activity* categories^[[32]](#footnote-32)^, and (ii) data describing the local, city-scale demography in terms of the population-split between these same categories.

The former data include, for each category of person, the spread of weekly spending across 150 products/sectors. Using the latter these can be converted to total, city-level spending by considering the number of people of each category residing in Bristol. Such demographic data is obtained from the local census and hence is available for all local government regions across the UK. The 150 products/sectors can then be matched up to the emissions intensity sectors of Eora and thus replace the national level HH-FD. Finally, it is necessary to estimate how much of this HH-FD is spent on domestic and imported good, respectively, and for this we assume the national-level splits of Eora are appropriate.

This discussion of data sources points to a number of potential areas of focus for future work. In particular, there are various national-level assumptions and data we use that would benefit by being replaced with local-level estimates. These included our simple, per-capita downscale of government and capital final demand and our use of national-level emissions intensities, domestic/imported splits of final demand, and household spending profiles for different economic actors. One modification that could be made relates to local average wages. Currently, we assume that persons’ spending profiles only differ by level of employment, but not by any city-specific factors relating to incomes. But in cities where average incomes are higher, consumption-based emissions are likely to be higher as well, albeit not linearly so^[[33]](#footnote-33)^. This could easily be changed by accounting for the ratio of Bristol- to national-level average incomes. However, while the carbon intensities we use remain national-level, there is a risk of double counting here: higher incomes in a particular area can reflect higher living costs that are not necessarily associated with greater emissions. Incorporating local-level data is therefore far from a trivial issue and remains a focus of our future work.

*Figure 6: Historical consumption-based emissions for Bristol split by source of final demand (production-based emissions are shown for comparison)*

1. <https://www.bristol.gov.uk/statistics-census-information> (accessed 25th April, 2016; as were all further links in the footnotes) [↑](#footnote-ref-1)
2. <https://data.gov.uk/dataset/national_travel_survey> [↑](#footnote-ref-2)
3. DECC 2014, Government GHG Conversion Factors for Company Reporting: Methodology Paper for Emission Factors,

   [http://www.ukconversionfactorscarbonsmart.co.uk](http://www.ukconversionfactorscarbonsmart.co.uk/) [↑](#footnote-ref-3)
4. <https://www.gov.uk/government/publications/road-transport-forecasts-2013> [↑](#footnote-ref-4)
5. *IPCC 2014,* Annex III: Technology-specific cost and performance parameters*. In: Climate Change 2014: Mitigation of Climate*

   *Change. Contribution of Working Group III to the Fifth Assessment Report of the Intergovernmental Panel on Climate*

   *Change* <http://www.ipcc.ch/report/ar5/wg3/> [↑](#footnote-ref-5)
6. Vehicle fleet composition projections <http://naei.defra.gov.uk/data/ef-transport> [↑](#footnote-ref-6)
7. Detailes on *The National Household Model* can be found here [www.cse.org.uk/projects/view/1233](http://www.cse.org.uk/projects/view/1233)

   *(accessed 6 March 2016)* [↑](#footnote-ref-7)
8. Investment Property Forum (2012) *Costing Energy Efficiency Improvements in Existing Commercial Buildings, IPF*

   *Research Programme 2011–2015,* [www.ipf.org.uk/resourcelibrary.html](http://www.ipf.org.uk/resourcelibrary.html) [↑](#footnote-ref-8)
9. Business Floorspace (Experimental Statistics) [www.gov.uk/government/statistics/business-floorspace-experimental-statistics](http://www.gov.uk/government/statistics/business-floorspace-experimental-statistics) [↑](#footnote-ref-9)
10. <https://data.gov.uk/dataset/domestic-energy-performance-certificates-lodged-on-register-by-energy-efficiency-rating> [↑](#footnote-ref-10)
11. IEA World Energy Investment Outlook 2014: Energy Efficiency Investment Assumption Tables, [www.worldenergyoutlook.org/investment](http://www.worldenergyoutlook.org/investment) [↑](#footnote-ref-11)
12. There may be other opportunities for efficiency improvements specific to certain industries, but the available data does not allow us to assess these. For our particularly case study of Bristol, where there are no significant steel or concrete plants for example, this omission is likely to have only small impacts upon the results [↑](#footnote-ref-12)
13. This GVA data is provided by the West of England Local Economic Partnership, and is output from the Area Economy Model produced by the RED Group at the University of Plymouth. [↑](#footnote-ref-13)
14. [www.ons.gov.uk/economy/environmentalaccounts](http://www.ons.gov.uk/economy/environmentalaccounts) [↑](#footnote-ref-14)
15. [www.gov.uk/government/publications/industrial-decarbonisation-and-energy-efficiency-roadmaps-to-2050](http://www.gov.uk/government/publications/industrial-decarbonisation-and-energy-efficiency-roadmaps-to-2050) [↑](#footnote-ref-15)
16. Gouldson et al. (2014) *The Economics of Low Carbon Cities: A Mini-Stern Review for Birmingham and the Wider Urban Area*, [www.lowcarbonfutures.org/reports/research-reports](http://www.lowcarbonfutures.org/reports/research-reports) [↑](#footnote-ref-16)
17. Vehicle fleet composition projections <http://naei.defra.gov.uk/data/ef-transport> [↑](#footnote-ref-17)
18. [www.gov.uk/government/publications/road-transport-forecasts-2011-results-from-the-department-for-transports-national-transport-model](http://www.gov.uk/government/publications/road-transport-forecasts-2011-results-from-the-department-for-transports-national-transport-model) [↑](#footnote-ref-18)
19. [www.theccc.org.uk/publications/](http://www.theccc.org.uk/publications/) [↑](#footnote-ref-19)
20. Andy Gouldson and Joel Millward-Hopkins (2015) *The Economics of Low Carbon Cities: A Mini-Stern Review for the City of Bristol*, [www.lowcarbonfutures.org/reports/research-reports](http://www.lowcarbonfutures.org/reports/research-reports) [↑](#footnote-ref-20)
21. Further details on the relationship between prices and demand can be found in DECCs methodology documents: [www.gov.uk/government/publications/updated-energy-and-emissions-projections-2014](http://www.gov.uk/government/publications/updated-energy-and-emissions-projections-2014) [↑](#footnote-ref-21)
22. Wiedmann (2009) *A review of recent multi-region input–output models used for consumption-based emission and resource accounting*, Ecological Economics, 69, 211-222 [↑](#footnote-ref-22)
23. Peters (2010) *Carbon footprints and embodied carbon at multiple scales,* Current Opinion in Environmental Sustainability, 2, 245-250 [↑](#footnote-ref-23)
24. Peters et al. (2012) *A synthesis of carbon in international trade*, Biogeosciences, 9, 3247-3276 [↑](#footnote-ref-24)
25. Peters (2008) *From production-based to consumption-based national emission inventories*, Ecological Economics, 65, 13-23 [↑](#footnote-ref-25)
26. Lenzen et al. (2013) *Building Eora: A Global Multi-Region Input-Output Database at High Country and Sector Resolution*, Economic Systems Research, 25, 20-49 [↑](#footnote-ref-26)
27. Kanemoto et al. (2014) *International trade undermines national emission reduction targets: New evidence from air pollution*, Global Environmental Change, 24, 52-59 [↑](#footnote-ref-27)
28. Committee on Climate Change (2013) *Reducing the UK’s carbon footprint and managing competitiveness risks*, London, UK. [↑](#footnote-ref-28)
29. Scott & Barrett (2015) *An integration of net imported emissions into climate change targets*, Environmental Science & Policy, 52, 150-157 [↑](#footnote-ref-29)
30. This has recently been extended for 1970 to 2011 [↑](#footnote-ref-30)
31. These are now referred to as the *Family Expenditure Surveys*: <https://data.gov.uk/dataset/family_spending> [↑](#footnote-ref-31)
32. These categories include *economically active* persons (*employees: part-time, employees: full-time, self-employed, un-employed*) and *economically inactive* persons (*full-time students, retired persons, other economically inactive*) [↑](#footnote-ref-32)
33. Hertwich & Peters (2009) *Carbon footprint of nations: A global, trade-linked analysis* Env. Sci. & tech. *43*(16), 6414-6420 [↑](#footnote-ref-33)
